# Loss of dysbindin-1, a risk gene for schizophrenia, leads to impaired group 1 metabotropic glutamate receptor function in mice

**DOI:** 10.3389/fnbeh.2015.00072

**Published:** 2015-03-26

**Authors:** Sanjeev K. Bhardwaj, Richard T. Ryan, Tak Pan Wong, Lalit K. Srivastava

**Affiliations:** Department of Psychiatry and Integrated Programme in Neuroscience, Douglas Mental Health University Institute, McGill UniversityMontreal, QC, Canada

**Keywords:** schizophrenia, animal model, cognition, memory, hippocampus, synaptic plasticity

## Abstract

The expression of dysbindin-1, a protein coded by the risk gene *dtnbp1*, is reduced in the brains of schizophrenia patients. Evidence indicates a role of dysbindin-1 in dopaminergic and glutamatergic transmission. Glutamatergic transmission and plasticity at excitatory synapses is critically regulated by G-protein coupled metabotropic glutamate receptor (mGluR) family members, that have been implicated in schizophrenia. Here, we report a role of dysbindin-1 in hippocampal group 1 mGluR (mGluRI) function in mice. In hippocampal synaptoneurosomal preparations from sandy (sdy) mice, that have a loss of function mutation in dysbindin-1 gene, we observed a striking reduction in mGluRI agonist [(S)-3, 5-dihydroxyphenylglycine] (DHPG)-induced phosphorylation of extracellular signal regulated kinase 1/2 (ERK1/2). This mGluR-ERK1/2 deficit occurred in the absence of significant changes in protein levels of the two members of the mGluRI family (i.e., mGluR1 and mGluR5) or in another mGluRI signaling pathway, i.e., protein kinase C (PKC). Aberrant mGluRI-ERK1/2 signaling affected hippocampal synaptic plasticity in the sdy mutants as DHPG-induced long-term depression (LTD) at CA1 excitatory synapses was significantly reduced. Behavioral data suggest that the mGluRI hypofunction may underlie some of the cognitive abnormalities described in sdy mice as the administration of CDPPB (3-cyano-N-(1, 3-diphenyl-1H-pyrazol-5-yl benzamide), a positive allosteric modulator of mGluR5, rescued short-term object recognition and spatial learning and memory deficits in these mice. Taken together, our data suggest a novel role of dysbindin-1 in regulating mGluRI functions.

## Introduction

The dystrobrevin binding protein 1 (*dtnbp1*) gene, coding for dysbindin-1 protein, was one of the first candidate risk genes reported for schizophrenia (Straub et al., 2002). Notably, genetic association studies reveal that variations in the DTNBP1 are associated with prefrontal cortical functions in schizophrenia patients as well as performances in episodic and working memories in healthy subjects (Burdick et al., 2007; Fallgatter et al., 2010; Zhang et al., 2010). The link between dysbindin-1 and cognition is interesting given that cognitive deficits in schizophrenia are widely considered to be the core symptoms for which no adequate treatment strategy is available (Kalkstein et al., 2010). The evidence from human genetic studies is strengthened by post-mortem data showing reductions in dysbindin-1 protein and mRNA in the hippocampus and prefrontal cortex (PFC) of schizophrenia brains (Weickert et al., 2008; Tang et al., 2009a). Dysbindin-1 is widely expressed in the brain; in the hippocampus, dysbindin-1 protein is expressed in the cell body, dendrites and spines of CA1, CA2/3 pyramidal neurons as well as in a subset of interneurons (Talbot et al., 2009).

In diverse cells, dysbindin-1 binds to proteins in a complex known as the biogenesis of lysosome-related organelle complex-1 (BLOC-1) which plays a role in protein trafficking in endosomal-lysosomal system (Li et al., 2003; Ghiani et al., 2010). Knowledge of *in vivo* neural functions of dysbindin-1 has mainly come from studies in sandy (sdy) mice that have a natural autosomal recessive mutation in *dtnbp1* gene causing a loss of dysbindin-1 protein expression (Swank et al., 1991). Studies from ours and other groups reveal that dysbindin deficiency in sdy mice leads to a number of cognitive and social behavioral impairments, e.g., deficits in spatial and working memory (Cox et al., 2009; Jentsch et al., 2009; Karlsgodt et al., 2011), object recognition memory (Bhardwaj et al., 2009), fear memory (Bhardwaj et al., 2009), and social interaction (Hattori et al., 2008). Evidence points to an important role of dysbindin-1 in dopaminergic as wells as glutamatergic synaptic transmission (Jentsch et al., 2009; Ji et al., 2009; Karlsgodt et al., 2011; Papaleo et al., 2012). For example, reductions in presynaptic glutamate release probability and a decrease in NMDA receptor mediated currents in the PFC of sdy mice have been reported (Tang et al., 2009b; Karlsgodt et al., 2011). On the other hand, in the hippocampus, NMDA NR2A-containing NMDA receptor mediated current and long-term potentiation (LTP) were found to be increased in sdy mice (Tang et al., 2009b). The behavioral significance of glutamatergic abnormalities in the dysbindin-deficient mice are, however, unclear.

Glutamatergic synaptic transmission is mediated by a set of fast ionotropic receptors (NMDA, AMPA and Kainate) as well as three families of G-protein coupled metabotropic glutamate receptors (mGluRI, II and III) (Ferraguti et al., 2008; Niswender and Conn, 2010). The mGluRI family, that includes closely related mGluR1 and mGluR5 subtypes are important slow-paced modulators of fast ionotropic glutamate receptors and synaptic plasticity (Gladding et al., 2009; Mukherjee and Manahan-Vaughan, 2013). Intracellular signaling by mGluRI family involves activation of classical Gq/G_11_- phospholipase Cβ pathway, resulting in the mobilization of intracellular calcium and activation of protein kinase C (PKC). In addition, mGluRIs also activate mitogen-activated protein kinase (MAPK) signaling including activation of extracellular signal regulated kinase 1/2 (ERK1/2) (Niswender and Conn, 2010). The mGluRI receptors are known to play important roles in learning, memory, and emotional behaviors (Conquet et al., 1994; Lu et al., 1997; Fowler et al., 2011).

A primary deficit in glutamate NMDA receptor-mediated transmission is the mainstay of glutamatergic hypothesis of schizophrenia (Coyle, 2012). However, the reported findings of altered mGluR1 expression in post-mortem schizophrenia brains and the clinical success of a group II mGluR agonist in the treatment of schizophrenia suggest a potentially important role of mGluRs in schizophrenia (Patil et al., 2007; Volk et al., 2010). Here, we worked on the hypothesis that the behavioral phenotype of dysbindin-deficient sdy mice is related to altered glutamatergic transmission through mGluRs. Our data show that dysbindin-1 deficiency leads to a marked reduction in a specific signaling pathway of mGluRI, and is associated with an abnormal hippocampal synaptic plasticity in sdy mice. Furthermore, enhancing mGluR5 function through a positive allosteric modulator (PAM) rescued object recognition and spatial learning and memory deficit of sdy mice, suggesting an important role of the group 1 mGluRs in the cognitive impairments caused by dysbindin-1 deficiency.

## Materials and Methods

### Materials

S-DHPG [(S)-3, 5-dihydroxyphenylglycine] and CDPPB (3-cyano-N-(1, 3-diphenyl-1H-pyrazol-5-yl) benzamide), were purchased from Tocris Bioscience (Ellisville, MO). Polyclonal antibodies against total-ERK 1/2 (Cat#9102) phospho-ERK1/2 (phosphorylated at Thr202 and Tyr204 of Erk1;Thr185 and Tyr187 of Erk2, Cat#9101), and phospho-PKC (Pan) (autophosphorylated at carboxy terminal Ser660 residue; Cat #9371) were from Cell Signaling (Beverly, Massachusetts, USA). Monoclonal antibody against mGluR1 was purchased from BD Transduction (Franklin Lakes, NJ). Polyclonal antibody for mGluR5 was obtained from Upstate Biotechnology (Lake Placid, NY). Antibody against the housekeeping protein β-actin was from Sigma (St. Louis, Missouri, USA). All other materials were purchased from commercial sources.

### Animals

The experiments, approved by the Animal Care and Use Committee of the Douglas Hospital Research Centre, were carried out in accordance with the guidelines of the Canadian Council of Animal Care. The sdy mutation, originally on DBA/2J strain (Swank et al., 1991) was transferred to C57 Black background by backcrossing with C57BL/6J mice for ten generations. Wild-type (WT) and homozygous mutant animals (sdy/sdy; hereinafter referred to as sdy) used in the present study were obtained by breeding of heterozygous parents and were littermates. Genotype was determined by genomic polymerase chain reaction (PCR) as reported by Cox et al. (2009). After weaning, male mice were group housed (4 animals/cage) in a semi enriched environment with 12 h:12 h light dark cycle with lights on at 8.00 am and had ad-libitum food and tap-water. Studies were conducted on age-matched adult (10–12 week old) male WT and sdy mice.

### Synaptoneurosomal Preparation and Drug Treatments

Synaptoneurosomes were prepared according to the procedure described by Kim et al. (2008). Briefly, the brains of mice were removed after decapitation, and the whole hippocampus immediately dissected out using thick coronal slices (500 μm). The tissue was homogenized using a glass-Teflon homogenizer in 50 mM Hepes, pH 7.5, 125 mM NaCl, 100 mM sucrose, 2 mM potassium acetate and 0.2 mM sodium orthovanadate. The homogenate was filtered through a series of nylon mesh filters (149, 62, and 10 microns; Small Parts Inc). The final filtrate was spun briefly (4,000 × *g*, 1 min) and the pellet was suspended in the homogenizing buffer. This suspension was incubated with 1 μM tetrodotoxin, on ice for 5 min, followed by 5 min at room temperature.

For mGluRI activation, synaptoneurosomal preparation from each animal (*n* = 5–8 per genotype) was incubated with vehicle (HEPES buffer) (no stimulation) or 5 μM of a specific mGluRI/5 agonist S-DHPG (hereinafter referred to as DHPG) for 2.5 min. At the end of incubation, samples were instantly lysed in 1.2% Triton x-100 and mixed with sample buffer (0.25 M Tris–HCl, pH 6.8, 20% glycerol, 4% SDS, 10% β-mercaptoethanol) for Western blotting using antibodies against signaling proteins ERK1/2 or PKC. In a separate cohort of WT and sdy animals, we assessed the time course of ERK1/2 activation by incubating synaptoneurosomes for 0, 1, 2.5 and 5 min with subsaturating (5 μM) and saturating (50 μM) concentrations of DHPG. To assess if the impaired ERK phosphorylation in sdy mice can be recovered by PAM of mGluR5, in another cohort of animals (*n* = 4 each genotype), the synaptoneurosomal preparations from WT and sdy mice were preincubated with vehicle (0.5% methylcellulose) or CDPPB (5 μM, 15 min), following which DHPG (5 μM) or vehicle was added and the samples incubated for a further 2.5 min. The samples were processed for Western blotting for p-ERK1/2 and β-actin as described below.

### Western Blotting

The procedures were essentially as described by us previously (Bhardwaj et al., 2014). The control and DHPG-treated synaptoneurosomal samples were electrophoresed and blot transferred onto nitrocellulose membranes (Hybond ECL, Amersham-Pharmacia Biotech). The membranes were incubated with 1:1000 dilution of rabbit polyclonal antibodies against phospho-ERK1/2, total-ERK 1/2 or p-PKC. Following washes in TBS–Tween-20, blots were incubated with anti-rabbit IgG-horseradish peroxidase (HRP)-conjugated secondary antibodies. To assess the expression levels of receptors mGluR1 and mGluR5, samples from the unstimulated synaptoneurosomes from WT and sdy mice (*n* = 5–6 per genotype) were Western blotted with antibodies against mGluR1 (1:2500), or mGluR5 (1:1000). The blots were developed using chemiluminescence detection system (Perkin-Elmer) and exposed to X-ray film (Biomax XAR, Kodak). To account for variations in protein loading, the blots were stripped in 62.5 mM Tris, 2% SDS and 100 mM β-mercaptoethanol (pH 6.7) for 30 min at 50°C and reprobed with a monoclonal anti-β-actin antibody (1:5000). Antibody-labeling intensity (relative optical density, ROD) was analyzed using a computerized image analysis system (MCID-4, Imaging Research) and results are expressed as ratio of RODs of specific protein to RODs of β-actin. The data were analyzed using 2-way analysis of variance (ANOVA) with genotype and DHPG treatment as independent variables followed by *post hoc* Tukey’s test (significance at *p* < 0.05). The data of receptor proteins (mGluR1 and mGluR5) in WT and sdy animals were compared using the student’s *t*-test.

### Electrophysiological Recordings

Electrophysiological experiments were done essentially as described by us previously (Bagot et al., 2012). WT and sdy male mice (*n* = 4–5), 50–60 day-old were decapitated and their brains were rapidly removed. Coronal slices containing the hippocampal area (350 μm) were sectioned using a Vibratome (Leica) in chilled (~0–4°C) oxygenated (95% O_2_/5% CO_2_) slice cutting solution containing (in mM) sucrose 252, KCl 2.5, MgCl_2_ 4, CaCl_2_ 0.1, KH_2_PO_4_ 1.25, NaHCO_3_ 26 and D-glucose 10. Freshly cut slices were placed in an incubating chamber with carbogenated artificial cerebrospinal fluid (ACSF) composed of the following (in mM): NaCl 125, KCl 2.5, NaHCO_3_ 26, NaH_2_ PO_4_ 1.25, MgCl_2_ 1, CaCl_2_ 2, and D-glucose 25 and recovered at 32°C for 1 h, followed by room temperature incubation in ACSF before and during recording. All recordings were performed in a submerged-type recording chamber that was perfused with ACSF containing GABA_A_ receptor antagonist bicuculline (10 μM) at a flow rate of 1.5 ml/min to block GABA_A_ receptor-mediated inhibitory synaptic transmission. Field excitatory postsynaptic potentials (fEPSPs) were evoked by stimulating the Schaffer collateral pathway (constant current pulses (0.08 ms) through a tungsten bipolar electrode (FHC) and recorded by a glass pipette filled with ACSF in the stratum radiatum of the CA1 region at least 60–80 μm away from the cell body layer. Synaptic responses were evoked at 0.05 Hz throughout, amplified and digitized by Multiclamp 700B and Digidata 1400 respectively (*Axon*), and stored in a PC for offline analysis using Clampfit (*Axon*). All recordings were performed at room temperature. Slices displaying unstable baseline recording were discarded. After obtaining a stable baseline for 30 min, LTD was induced by a group I mGluR agonist DHPG. (37.5 μM, applied for 10 min).

### Novel Object Recognition (NOR) Memory

Our previously described procedure was followed (Bhardwaj et al., 2009). Briefly, WT and sdy mice (*n* = 7–8/genotype) were acclimatized for 20 min × 3d to a dark Plexiglas open-field chamber (*L* × *W* × *H*: 45 × 45 × 45 cm) kept in a dimly lit and noise-free room. NOR testing began next day by injecting the mice with vehicle (0.5% methylcellulose, i.p.) or the mGluR5 PAM CDPPB (10 mg/kg, i.p.). Thirty minutes later the animals were introduced in the chamber with two identical objects (small platic toys of various shapes, Dollar Store). The dose of CDPPB was based on previous reports on the efficacy of CDPPB in cognitive tasks (Uslaner et al., 2009). The animals were allowed to explore the objects for 5 min (sample phase) after which they were placed back in their respective home cages for 5 min (retention time for short-term memory). At the end of retention time, the animals were re-introduced to the chamber containing a novel object and a new copy of the familiar object used during the sample phase. Animals were allowed to explore the objects for 5 min (testing phase) and then returned to the home cage. To test longer-term NOR memory, 24 h later, the mice again went through similar sample and testing phases with a different novel object and a new copy of the familiar object used during the previous day’s sample phase. Behaviors during the sample and testing phases were videotaped with an overhead recording camera and the videos were scored for the time spent by the animals exploring the two objects. A mouse was considered to be involved in object exploration when its head was oriented directly towards the object and within approximately 2–3 cm from it. The time was also included if animal had at least one forepaw on the object or was sniffing or licking the object. Object recognition memory was defined as the ratio of exploration time for the novel object (*T_N_*) over exploration time for the novel plus familiar object (*T_F_*) (exploration ratio = *T_N_*/(*T_F_* + *T_N_*)). Recognition memory above chance level (0.5) was analyzed by “one-sample *t*-test” of the exploration ratio data. Group differences in the exploration ratio as well as the total time spent exploring both objects were analyzed using 2-way ANOVA with genotype and drug treatments as independent variables, followed by, if indicated, Tukey’s *post hoc* test (significance at *p* < 0.05).

### Spatial Learning and Memory in Morris Water Maze (MWM)

A new cohort of WT and Sdy animals (*n* = 6–7 each group) was used in this experiment. Our previously described MWM procedure, modified for mice was employed to assess the effect of CDPPB on spatial learning and memory (Wood et al., 2003). The MWM for mice mouse water maze, consisting of a circular pool (1.4 m diameter and 36 cm high) with a removable plexiglass platform (10 × 10 cm), was placed in a room with various distal visual cues and a ceiling-mounted video recording camera. The platform was hidden by the pool water (at 22–24°C) made opaque with a non-toxic paint. Animals were administered saline or CDPPB (10 mg/kg, i.p.) and 30 min later released in different quadrants of the pool in a pseudorandom order. Animals’ latency to find the fixed and submerged platform was analyzed by Water 2100 software. Each animal was given three trials per day with an ITI of 1 h. Each trial lasted for 60 s unless the animal reached the platform before. These spatial learning trials were given for six consecutive days with drug treatment each day, and the latency data was averaged. Two hours after the last trial on the final day of training, spatial memory was assessed in the pool without the platform with 3 trials of 60 s each from a different start location (probe test). The time spent in the target quadrant and annulus crossing of the platform location was analyzed. At the end of probe trials, the platform was raised above the pool water and the animals’ latency to find the visible platform and swim speed were assessed for potential visual or locomotor impairments. Spatial learning and probe test data were analyzed using 3-way repeated measure and 2-way ANOVA respectively. Where indicated, Tukey’s *post hoc* was used to determine significant differences (*p* < 0.05).

## Results

### Levels of mGluR1 and mGluR5 Proteins in Hippocampal Synaptoneurosomes

**Figure 1:**
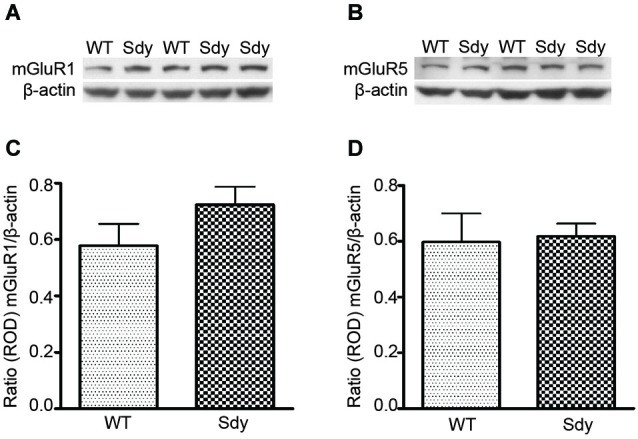
**Western blot analysis of hippocampal mGluR1 and mGluR5 protein levels show no significant differences between wildtype (WT) and dysbindin deficient (sdy) animals**. Top panel show representative immunoblots of mGluR1 **(A)**, mGluR5 **(B)** and respective β-actin expression. Lower panel shows relative optical density (ROD) ± SEM (*n* = 5–6 per genotype) of mGluR1 **(C)** and mGluR5 **(D)** normalized to β-actin levels.

Figure 1 shows representative Western blots and analyses of protein levels of mGluR1 and mGluR5. ROD data (normalized to β-actin) revealed no significant differences in either mGluR1 or mGluR5 levels between WT and sdy mice (Student’s two-tailed *t*-test *t*_(9)_ = 1.471, *p* = 0.175 and *t*_(9)_ = 0.192, *p* = 0.852 respectively for mGluR1 and mGluR5).

### Effect of mGluRI Activation on ERK1/2 and PKC

**Figure 2:**
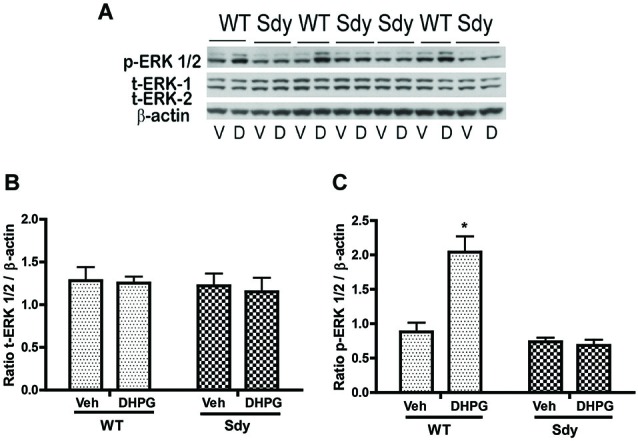
**mGluR1-induced ERK1/2 phosphorylation in hippocampal synaptoneurosomes in WT and sdy mice**. Synaptoneurosomes were incubated with a group I mGlu receptor agonist S-DHPG (5 μM) for 2.5 min at room temp. **(A)** shows the representative Western blot signals using antibodies specific for p-ERK 1/2, (total) t-ERK 1/2 and β-actin. DHPG stimulation triggered significant ERK1/2 phosphorylation in WT but not in sdy synaptoneurosomes. (V, vehicle; D, DHPG). **(B,C)** show ROD ± SEM of t-ERK1/2 and p-ERK1/2 respectively (*n* = 5–8 per genotype). *p = 0.0015 WT DHPG vs. WT vehicle (Tukey’s *post hoc*).

**Figure 3:**
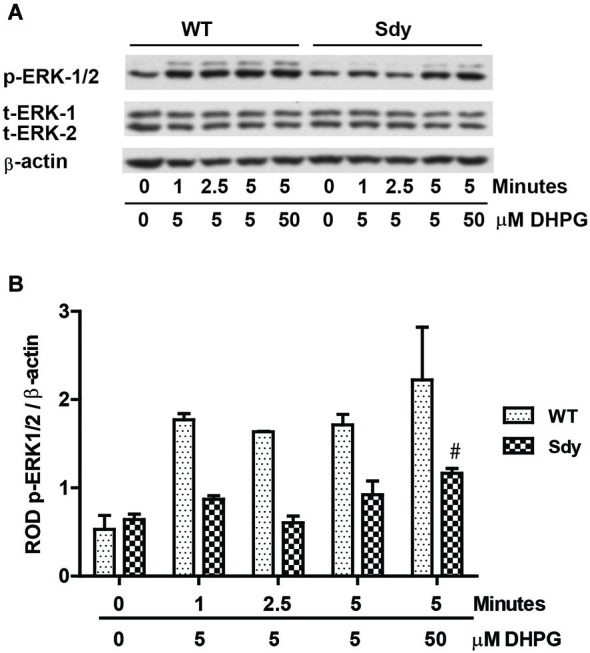
**Impaired ERK1/2 activation by mGluRI stimulation is observed rapidly at both sub-saturating (5 μM) and saturating (50 μM) concentration of mGluRI agonist S-DHPG. (A)** shows the representative Western blot signals of p-ERK1/2, total (t)-ERK 1/2 and β-actin. **(B)** shows the histogram of ROD of mean ± SEM (*n* = 6 per genotype) of the normalized p-ERK1/2.

**Figure 4:**
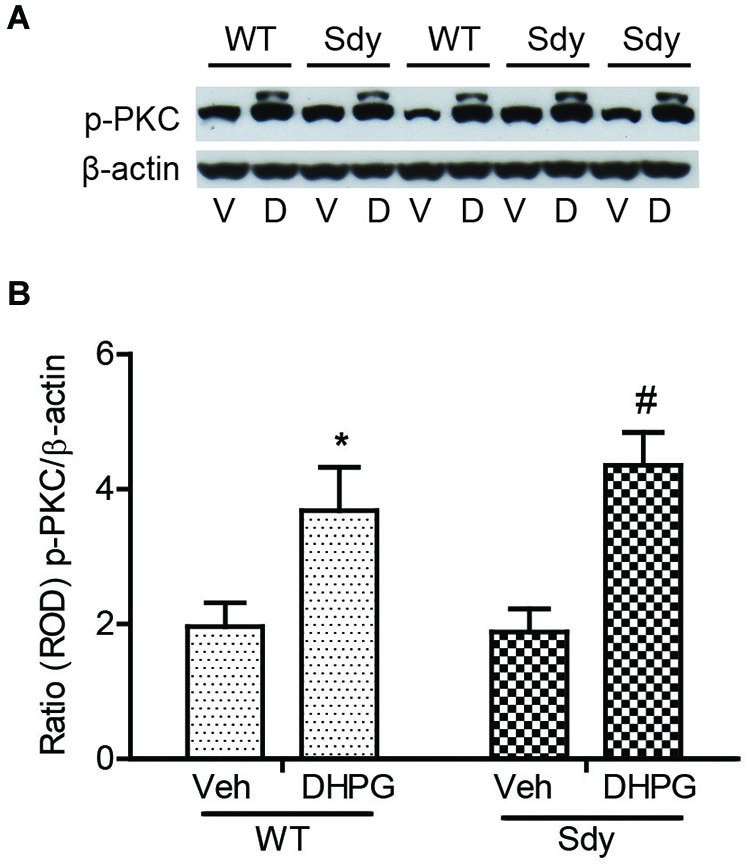
**No significant change in mGluR1-induced hippocampal PKC phosphorylation between WT and sdy mice**. Synaptoneurosomes were incubated with 5 uM DHPG for 2.5 min at room temp. Phosphorylated PKC (p-PKC) was measured by Western blots, using a pan p-PKC antibody. **(A)** representative Western blot of the expression of phospho-PKC (p-PKC) and β-actin from WT and Sdy mice (V, vehicle; D, DHPG). **(B)** mean + SEM (*n* = 4–6 per genotype) of the ratio from p-PKC vs. β-actin. Two-way ANOVA indicated the significant main effect of treatment only (*p = 0.032 WT DHPG vs. WT vehicle; #:*p* = 0.009 sdy DHPG vs. sdy vehicle).

The levels of total and DHPG-activated ERK1/2 and PKC were measured in hippocampal synaptoneurosomal preparations. Figure 2A shows the representative Western blots and analyses of total and phospho ERK1/2 levels in WT and sdy mice. A two-way ANOVA showed no significant difference in the levels of total ERK1/2 between WT and sdy animals either at the basal level (i.e., vehicle) or after DHPG incubation (genotype: *F*_(1,24)_ = 0.0057, *p* = 0.981; DHPG treatment: *F*_(1,24)_ = 0.099, *p* = 0.756; genotype × DHPG interaction: *F*_(1,24)_ = 0.042, *p* = 0.839) (Figure 2B). However, analysis of P-ERK1/2 data showed significant main effects of genotype (*F*_(1,24)_ = 34.13, *p* = 0.0001), DHPG treatment (*F*_(1,24)_ = 18.60, *p* = 0.0002) and genotype × DHPG interaction (*F*_(1,24)_ = 22.22, *p* = 0.001) (Figure 2C). As expected, DHPG induced significant increase in P-ERK 1/2 in WT synaptoneurosomes; however, DHPG-induced ERK1/2 phosphorylation was markedly attenuated in the sdy animals. Impaired ERK1/2 phosphorylation in sdy synaptoneurosomes was seen rapidly. For example, the difference in p-ERK1/2 between WT and sdy animals is observed as early as 1 min after DHPG application and persisted when saturating concentrations of DHPG (50 μM) were used. A two-way ANOVA of the data revealed a significant effect of genotype (*F*_(1,20)_ = 30.66, *p* = 0.0001), DHPG concentration (*F*_(4,20)_ = 7.47, *p* = 0.0001) and genotype × DHPG interaction (*F*_(4,20)_ = 2.66, *p* = 0.042) (Figure 3). *Post hoc* test showed that ERK1/2 phosphorylation in Sdy mice increased at 50 μM DHPG compared to 5 μM (*p* = 0.046) suggesting that increasing the concentration of DHPG may overcome ERK1/2 deficits in sdy mice. In order to find out if mGluRI signaling deficit in sdy mice is specific to ERK1/2 pathway, we measured the level of phospho-PKC in synaptoneurosomes incubated with DHPG. The data showed a significant effect of DHPG treatment on PKC phosphorylation (*F*_(1,16)_ = 19.66, *p* = 0.0004); however, no significant effect of the genotype (*F*_(1,16)_ = 0.39, *p* = 0.539) or genotype × DHPG interaction (*F*_(1,16)_ = 0.63, *p* = 0.437) was observed (Figure 4). In order to investigate if deficits in ERK1/2 phosphorylation in sdy animals are ameliorated by mGluR5 PAM CDPPB, synaptoneurosomes were incubated with Vehicle, DHPG (5 μM), CDPPB (5 μM) or CDPPB (5 μM) + DHPG (5 μM). Two way ANOVA of p-ERK1/2 data shows a significant effects of genotype (*F*_(1,24)_ = 12.08, *p* = 0.002), drug treatment (*F*_(3,24)_ = 16.93, *p* = 0.0001) and genotype × drug interaction (*F*_(3,24)_ = 3.89, *p* = 0.021). *Post hoc* showed significantly increased DHPG-induced p-ERK1/2 in Sdy synaptoneurosome preincubated with CDPPB. This effect of CDPPB was not observed in the WT synaptoneurosomes (Figure 8).

**Figure 5:**
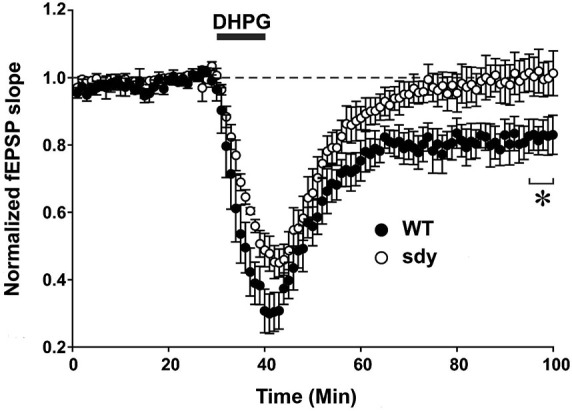
**Scatter plots show decreased LTD of field excitatory postsynaptic potential (fEPSP) induced by mGluRI agonist S-DHPG**. Hippocampal slices were incubated with 37.5 μM DHPG applied for 10 mins. Recordings were done in a chamber perfused continuously by carbogenated ACSF containing bicuculline methiodide (10 μM) to block GABAA receptor-mediated inhibitory synaptic currents. fEPSPs were evoked by stimulating the Schaffer collateral-commissural pathway via a constant current pulse (0.08 ms) delivered through a tungsten bipolar electrode (FHC) at 0.05 Hz throughout and recorded by a glass pipette filled with ACSF in the stratum radiatum of the CA1 region at least 60–80 μm away from the cell body layer. Slices displaying unstable baseline recording were discarded. After obtaining a stable baseline for 10 min, LTD was induced by S- DHPG. (*p < 0.05, comparison of the average fEPSP slope between WT and sdy mice, (*n* = 4–5), Student’s *t* test).

**Figure 6:**
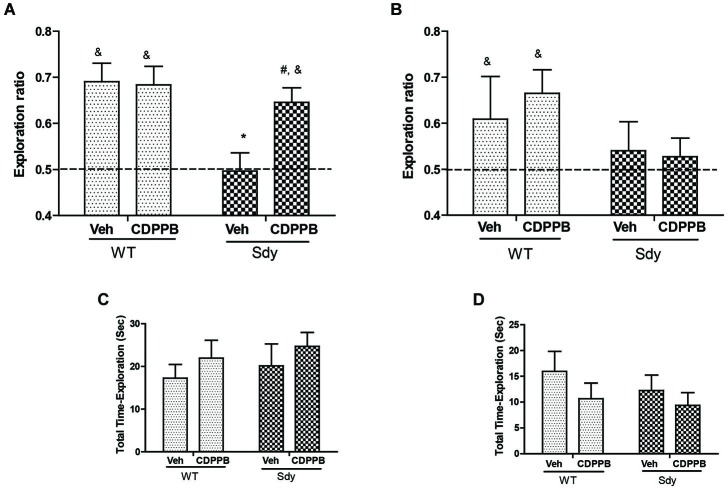
**NOR by WT and dysbindin mutants (sdy) animals following 5 min and 24 h retention intervals**. Both WT and sdy animals were injected with either vehicle (Veh) or CDPPB (10 mg/kg, ip) (*n* = 7–8/genotype/treatment), 30 min before the sample phase. In the vehicle treated groups, novel object exploration ratio following 5 min retention interval **(A)** indicates that sdy mice show less preference for the novel object compared to WT controls (*p = 0.001). Administration of CDPPB resulted in the rescue of NOR deficits in sdy mice (#*p* = 0.009 compared to sdy vehicle group) **(A)**. At longer retention time (24 h), CDPPB administration had no significant effect **(B). (C,D)** show that the total exploration time of objects at 5 min and 24 h retention intervals respectively are not significantly different between the groups.

**Figure 7:**
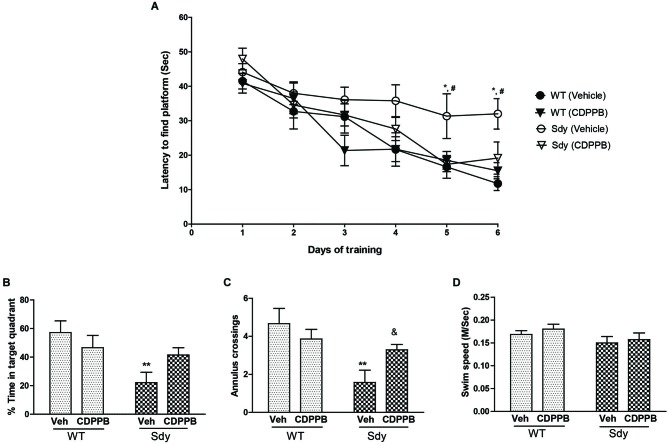
**Spatial learning and memory in MWM**. WT and sdy animals were injected with either vehicle (Veh) or CDPPB (10 mg/kg, ip), (*n* = 6–7 per genotype/treatment) once a day, for 6 days, 30 min before the first trial of the day in. **(A)** Escape latency (seconds) during hidden platform location for WT and sdy animals following either vehicle or CDPPB administration. Sdy-vehicle animals have significant deficit in spatial learning compared to WT-vehicle animals on days 5 and 6 (*p < 0.05). CDPPB treatments significantly improved the learning deficit in sdy animals compared to vehicle treated sdy animals (#*p* < 0.05). **(B)** Time spent in the target quadrant in the probe test. Vehicle treated sdy mice showed a significant deficit compared to vehicle treated WT mice in the time spent in correct quadrant (**p = 0.002). CDPPB treatment attenuated this spatial memory deficit in sdy mice as no significant differences in the target quadrant preference was observed between sdy and WT CDPPB treated animals. **(C)** Average annulus crossings during probe trial for all animals. Similar to target quadrant preference, annulus crossings also revealed a significant deficit in spatial memory in sdy mice and its amelioration by CDPPB (**p = 0.0012, vehicle treated sdy mice compared to vehicle treated WT mice; (^&^*p* = 0.042, sdy CDPPB vs. sdy vehicle. **(D)** Visible platform data revealed no significant differences in mean swim speed among experimental groups.

**Figure 8:**
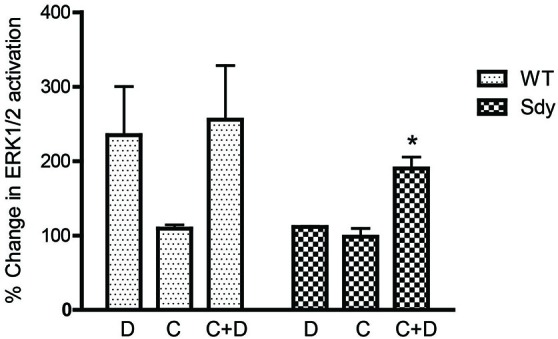
**Pretreatment with CDPPB partially normalizes DHPG-induced ERK1/2 deficit in Sdy mice**. Percent increase in βactin-normalized p-ERK1/2 in synaptoneurosomes incubated with CDPPB (C, 5μM, 15 min) without or with DHPG (D, 5μM 2.5 min) (*p = 0.043 compared to Sdy DHPG; *n* = 4 per genotype).

### mGluRI-Dependent LTD

Group 1 metabotropic glutamate receptors regulate excitatory synaptic plasticity, in particular an NMDAR-independent form of LTD (mGluRI-LTD) at CA1 excitatory synapses (Mukherjee and Manahan-Vaughan, 2013). As ERK1/2 signaling pathway is specifically implicated in this synaptic plasticity function of mGluRI (Gallagher et al., 2004), we asked whether mGluRI hypofunctionality observed in sdy mice is also expressing at the synaptic level. We compared DHPG-induced changes in fEPSP recorded from the CA1 region between slices from WT and sdy mice (Figure 5). While DHPG produced a robust LTD in WT mice slices (percent depression of fEPSP slope at 60 min after DHPG application: 18.1 ± 4.5%), it failed to induce significant LTD in the sdy mice slices (0.3 ± 4.7%; WT vs. sdy: *p* = 0.045, *Student’s *t*-test*).

### Effect of mGluR5 Positive Allosteric Modulator CDPPB on NOR

As studies show that enhancing mGluR5 transmission through PAMs such as CDPPB, DFB and ADX47273 improve cognitive functions and produce antipsychotic like effects in preclinical models (Conn et al., 2009), we examined whether CDPPB-induced enhancement of mGluR5 will ameliorate NOR memory in sdy mice. Our previous data had shown a significant deficit in short-term NOR memory in sdy mice (Bhardwaj et al., 2009) which was confirmed in the present study. The present results also show that pre-training administration of CDPPB prevented short-term (5 min) object recognition memory deficit in sdy mice (Figure 6). Two-way ANOVA of novel object preference ratio indicated a significant main effect of genotype (*F*_(1,28)_ = 9.39, *p* = 0.004) and genotype × CDPPB interaction (*F*_(1,28)_ = 4.25, *p* = 0.048) in 5 min memory test (Figure 6A). *Post hoc* tests confirmed the NOR deficit in sdy mice compared to the WT (*p* = 0.001). CDPPB treatment had no significant effect in WT mice; however, CDPPB administered sdy mice showed significantly better NOR performance compared to vehicle treated sdy animals (*p* = 0.009). One-sample *t*-test of the data also indicated that WT animals (both after vehicle or CDPPB treatment) and sdy with CDPPB treatment preferentially explore the novel object (significantly above 0.5 chance level). ANOVA of 24 h NOR data showed no significant effect of either genotype (*F*_(1,27)_ = 2.68, *p* = 0.11) or genotype × CDPPB interaction (*F*_(1,27)_ = 0.30, *p* = 0.58) (Figure 6B). One-sample *t*-test indicated a lack of significant effect of CDPPB on longer-term NOR deficit in sdy mice. No significant effect of the genotype or drug treatment was observed in the total exploration time of the objects in both short and long-term NOR tests (Figures 6C,D), indicating that the changes in NOR performance in sdy mice or the effects of CDPPB are not due to the altered general activity. We also separately confirmed the lack of effect of CDPPB on locomotor activity by beam breaks in activity boxes fitted with infra-red beams and sensors (Data not shown).

### Effect of CDPPB on Spatial Learning and Memory

A 3-way repeated measure ANOVA of spatial learning trials showed a significant genotype × CDPPB × days interaction (*F*_(5,115)_ = 2.31, *p* = 0.04). *Post hoc* tests showed that sdy animals have significant deficit in spatial learning as revealed by a significantly higher latency to reach platform compared to WT animals (Figure 7A). CDPPB treatments significantly improved the learning deficit in Sdy animals as their latency across days of trials was not significantly different from the WT animals. Analysis of spatial memory data (Figures 7B,C) showed a significant genotype × CDPPB treatment interaction (Probe test: *F*_(1,23)_ = 4.59, *p* = 0.04; Annulus crossing: *F*_(1,23)_ = 4.80, *p* = 0.038). *Post hoc* tests confirmed spatial memory deficit in sdy mice (sdy spent 22% of time in the target quadrant compared to 57% of time spent by WT mice, *p* = 0.002). CDPPB administration attenuated this spatial memory deficit as no significant differences in the target quadrant preference or annulus crossings were observed between sdy and WT after CDPPB treatments. Finally, swim speed data in visible platform test showed that genotype or CDPPB treatments had no significant effect on visual or motor abilities of the mice (Figure 7D).

## Discussion

One of the principal findings of our study is that a loss of function mutation in *dtnbp1*, a gene implicated in schizophrenia, in mice leads to impaired intracellular signaling and synaptic plasticity mediated by hippocampal group 1 metabotropic glutamate receptors. The hypofunctionality of mGluRIs are related to cognitive impairments due to dysbindin-1 deficiency, as a PAM of mGluR5 was found to attenuate object recognition and spatial learning and memory deficits in sdy mice. While previous studies suggested a role of dysbindin-1 in NMDA receptor function (Tang et al., 2009b; Karlsgodt et al., 2011), our study expands the link between dysbindin-1 and glutamatergic transmission to include mGluRI family.

Our data show that the decrease in ERK1/2 activation by a specific mGluRI agonist in sdy hippocampus occurs in the absence of significant changes in total protein levels of ERK1/2, levels of mGluR1 or 5 proteins or alterations in basal or agonist-induced PKC phosphorylation. The blunted ERK1/2 response appears to be due to a reduced affinity of mGluRI for the agonist, as increasing the concentration of DHPG partially ameliorated the deficit. These findings suggest the possibility of a role of dysbindin-1 in facilitating intracellular mGluRI receptor-effector coupling. We do not know whether ERK1/2 deficit is specific to the mGluR1 or the mGluR5 subtype of mGluRI family; the resolution of this will depend on the availability of specific agonists for mGluR1/mGluR5. However, given the steep reduction in DHPG-induced ERK1/2 and the predominant expression of mGluR5 subtype in the hippocampus (Romano et al., 1996), we believe that at least a part of this deficit in sdy animals, is due to mGluR5. We also do not know how the loss of dysbindin-1 may induce changes in mGluRI signaling. Dysbindin-1 functions are known to be mediated by binding to several protein partners involved in membrane trafficking and vesicle transport process (Guo et al., 2009). Whether dysbindin-1 interacts with mGluR1/5 proteins and modulates affinity of glutamate for the receptor, or interacts with G-proteins or signaling proteins upstream of ERK1/2 activation (e.g., src, Ras, Rap1 or MEK) and affects receptor-effector coupling are possibilities that remain to be explored. In regard to the possibility of protein-protein interactions between dysbindin-1 and mGluRI components, it is notable that dysbindin-1 is present at excitatory neuronal dendritic shafts and spines, sites of mGluR1 and 5 expression (Talbot et al., 2009).

A related possibility is that dysbindin-1 mutation affects membrane trafficking and endocytosis of mGluRIs in ways that reduces surface expression of the receptor and/or uncouples the receptor from specific signaling components (Dhami and Ferguson, 2006). A role of dysbindin-1 in the endocytosis and recycling of certain neurotransmitter receptors has been previously suggested using dysbindin mutants and cell cultures. For example, dysbindin-1 deficiency promotes diversion of dopamine D2 receptor and NR2A subunit of NMDA receptor from lysosomes to the cell surface (Ji et al., 2009; Tang et al., 2009b). Since group I mGluRs are known to undergo both agonist-dependent and -independent endocytosis in both heterologous cell expression systems and primary neuronal cultures (Dhami and Ferguson, 2006), it is possible that dysbindin-1 modulates recycling of this receptor system as well.

Our data show that impaired mGluRI-ERK1/2 signaling is functionally relevant as it reduces mGluRI-dependent long-term depression at the hippocampal CA1 excitatory synapses of sdy mice. Our data in WT mice is consistent with the reports that one of the most robust actions of the mGluRI agonist DHPG is the induction of LTD of excitatory postsynaptic potentials in CA1 pyramidal neurons (Gladding et al., 2009; Mukherjee and Manahan-Vaughan, 2013). However, it should be noted that group I mGluRs have also been implicated in LTP formation in both pyramidal neurons and interneurons in the hippocampus (for review, see Anwyl, 2009). Since the induction of LTD by DHPG is known to involve the signaling pathway of ERK1/2 MAP kinase (Gallagher et al., 2004), we hypothesized that hippocampal DHPG-induced LTD will be significantly reduced in dysbindin mutants because of potential receptor uncoupling. The results support our hypothesis since we found that DHPG-induced LTD at CA1 was smaller in magnitude and shorter in duration in sdy mice than WT controls. Our findings also underscore the point that the impact of sdy mutation could be specific to the mGluRI-mediated form of LTD, since the NMDAR-mediated LTD was reported to be unaffected in sdy mice (Tang et al., 2009b). In the same study, Tang et al. have shown that NMDAR-mediated LTP was facilitated in sdy mice, which suggests enhanced hippocampus-dependent cognitive functions in these mice. Notably, mGluRI-LTD is implicated in hippocampus-dependent cognitive processes such as novelty encoding, object place configurations and recognition (Braunewell and Manahan-Vaughan, 2001; Kemp and Manahan-Vaughan, 2004; Popkirov and Manahan-Vaughan, 2010). Our findings that sdy mice displayed impairment in most hippocampus-dependent cognitive tasks therefore support the importance of intact mGluR-LTD for these cognitive processes.

We focused on NOR and spatial earning in MWM as performance in these behaviors requires hippocampal network, and they are relevant to cognitive phenotype of schizophrenia. They are components of preclinical assays suggested by the Measurement and Treatment Research to Improve Cognition in Schizophrenia (MATRICS) initiative (Young et al., 2009). Schizophrenia patients show deficits in a number of cognitive domains including working declarative and visuso-spatial memory that can be partially modeled in rodents. NOR task models certain components of declarative memory in rodents by measuring spontaneous preference of rodents to explore a novel object (Ennaceur, 2010), whereas the MWM task interrogates hippocampal-dependent visual learning and memory (D’Hooge and De Deyn, 2001). Our findings of amelioration of object recognition and spatial learning and memory deficits in sdy mice by pre-treatment with mGluR5 PAM CDPPB suggest that group 1 GluR abnormality contributes, at least in part, to cognitive dysfunctions caused by dysbindin deficiency. Our data show that pre-training acute administration of CDPPB, rescued short-term (5 min) but not long-term (24 h) memory deficit in sdy mice. The differential effect of CDPPB on short vs. longer-term object recognition memory deficit could be related to the dose of the drug used or its plasma half-life (approximately 4 h). For MWM learning, animals were pretreated with CDPPB every day before putting them in the maze. Thus, the long-term memory improvement in this task appears be due to a chronic effect of CDPPB on learning performance. These data highlight differential acute vs. chronic effects of CDPPB and support a growing number of evidence that mGluR5 PAMs have robust efficacy in a range of learning and memory in preclinical models of schizophrenia (Liu et al., 2008; Conn et al., 2009; Homayoun and Moghaddam, 2010).

Our studies show that the dose of CDPPB used (10 mg/kg, i.p.), while improving object recognition and spatial memory in sdy animals, has no significant effect in the WT mice. This lack of effect of CDPPB in control mice is apparently inconsistent with previous studies that show improvement in object recognition in rats and spatial learning in mice by 3 and 30 mg/kg (i.p.) CDPPB respectively (Uslaner et al., 2009; Fowler et al., 2013). Thus, it seems that the effect of this mGluR5 PAM is dependent on the dose of the compound used, animal species and the behavioral task in question. The mechanism by which CDPPB attenuated memory deficits in sdy mice is not addressed in our studies. CDPPB is an allosteric activator of mGluR5 and enhances the efficacy of the physiological level of glutamate on mGluR5 receptor (Niswender and Conn, 2010). Since we noticed an enhancement of DHPG-induced p-ERK1/2 levels in Sdy hippocampus, we believe that the mGluRI-ERK1/2 deficit in sdy is possibly due to reduced agonist affinity of the receptors. Since ERK1/2 has been implicated in mGluR1/5-dependent LTD (Gallagher et al., 2004), we speculate that the rescue of behaviors in sdy mice by CDPPB might be due to normalized LTD. However, this idea needs to experimentally tested.

Several genetic variations associated with schizophrenia map on to cognitive phenotypes; however, the mechanisms of genetic abnormality and cognition are poorly understood. Metabotropic glutamate receptors have been implicated in schizophrenia and impaired mGluR1/5 function leads to a number of learning and memory deficits (Conquet et al., 1994; Lu et al., 1997). We believe that our novel finding that dysbindin-1-deficiency leads to a behaviorally significant state of mGluRI hypofunction provides a link between dysbindin-1 gene variations, glutamatergic transmission and cognitive dysfunctions in schizophrenia.

## Conflict of Interest Statement

The authors declare that the research was conducted in the absence of any commercial or financial relationships that could be construed as a potential conflict of interest.

## Abbreviations

CDPPB, 3-cyano-N-(1, 3-diphenyl-1H-pyrazol-5-yl benzamide; ERK1/2, extracellular signal regulated kinase 1/2; DHPG, [(S)-3, 5-dihydroxyphenylglycine]; mGluR, metabotropic glutamate receptor.
